# An Inquiry into the Nature of the Disease Called Milk-sickness

**Published:** 1852-05

**Authors:** L. P. Yandell

**Affiliations:** Louisville, Ky.


Art. II.—An Inquiry into the Nature of the Disease called Milk-sickness. By L. P. Yandell, M. D.

Since an early period in the history of the Western States a disease of a fatal character has been frequently described as occurring, from time to time, in particular localities. The subject has excited deep interest, and large rewards have been offered by several of the states for the discovery of the poison which produces the disease. A great many papers have been written concerning it, but up to the present hour, it is safe to affirm, no progress has been made towards the detection of the cause, and it has at length become a grave question with many, *whether there be such a disease as "Milk-sickness."* I confess that I am of the number of those who have been forced to doubt whether the disease described as Milk-sickness has any connection with poisoned milk, or the flesh of poisoned animals. One of my earliest public efforts was an essay on Milk-sickness, and since that time I have had occasion to read many curious speculations as to the cause of the malady. No one could have been more fully persuaded of the reality of the affection than I was when I wrote; but that confidence has been seriously shaken by the contradictory testimony since elicited in the discussion of the subject. I propose now to submit an analysis of so much of that testimony as can be given within convenient limits.

*Topography.*—Milk-sickness is reported to be an endemic of Ohio, Indiana, Illinois, Kentucky, Tennessee, Alabama, Mississippi, North Carolina, Georgia, and perhaps one or two other States. We will look, first, at what has been said about the physical aspect of the countries in which it occurs, and my first quotations will be from the inaugural dissertations of two of the recent graduates of the University of Louisville—Dr. Smydth and Dr. Armstrong, of Indiana. The former gentleman says: "Some who have written on this subject appear to be of opinion that Milk-sickness is a disease almost wholly confined to mountainous regions; but according to my observation it often prevails in level countries. The face of the country in that part of Indiana where it is most prevalent, is marked by ranges of hills, valleys, and plains, and I have met with cases of it in the latter. The soil for the most part is a loam, but in some places is composed chiefly of sand. Lime-stone is scarce where the disease prevails, the strata consisting of sand-stone, intermixed with coal and iron ore."

Dr. Armstrong says: "All modern writers agree, I believe, concerning the physical aspect of the country where Milk-sickness is found. It is generally hilly, the underlying rock being a sand-stone or black slate."

Dr. Seaton, in a pamphlet on this subject published eleven years since, says that "pure lime-stone regions are exempt" from the disease, and that "while it may prevail where the formation is a mixed one of lime-stone and sand-stone, the pure sand-stone formation connected with the ores of metals, is the distinguishing geological feature of those regions where the disease is most abundant."

Hartsville, around which Milk-sickness has been known for half a century, is in the midst of one of the most fertile regions in the world. The formation is lime-stone, of the age of that which underlies the country about Lexington and Cincinnati; but the "*Mill-Stone Knob*" whence the poison is supposed to be derived corresponds to the Silver creek hills, near Louisville, and is composed of black slate and sand-stone, chiefly the latter. It rises to an altitude of about 300 feet, and its summit is still covered with cane. It is on the sides of this knob that cattle are believed by the people to find the poison, but instances are related in which animals confined to pastures several miles remote from the knob, in the rich blue-grass lands below, have contracted it and died.

In North Carolina, the disease prevails, according to Dr. McCall, "on the lowlands of Yadkin river," as it prevails among the slashes of Mad river in Ohio. In Tennessee, it is met with either on mountains or hills, or in the valleys beneath, but when it occurs in the latter places it is generally ascribed by the people to a poison contracted by their cattle on the high lands. The legislature of Tennessee passed an act, in the year 1821, requiring fences to be made around certain coves of the Cumberland mountain, in Franklin county, "to prevent animals," as the act reads, "from eating an unknown vegetable, thereby imparting to their milk and flesh qualities highly deleterious." The barrier erected according to the provisions of this act was twelve or fifteen miles long.* The principal other localities of Milk-sickness in Tennessee are of the same physical character. They are ranges of hills, or valleys amid the mountains; but whether the disease originates on the elevated lands or in the valleys is a question not decided. Smith and Bedford counties, in which it is occasionally met with, are both broken. Some of the other points spoken of in connection with the disease are the Sequachee valley, and the valleys along a few of the tributaries of French Broad, Clinch, and Emory rivers. In Monroe and Blount, both mountainous counties, some of the farmers profess to have determined the limits of the poison. Says Dr. Shelton, referring to the former county, "there is a locality embracing not more than ten or fifteen acres, on which the disease has been known to originate for nearly, perhaps quite, forty years." He mentions a similar limited district in Monroe county, and goes on to say; "A third site occurs on the Chattahoochee river, in Georgia. It is an elevated piece of bottom land, containing scarcely more than five acres, shut in by the river, and by bluffs on every side."†

Dr. Drake, in the fall of 1840, visited one of the districts in Ohio "which has long had the reputation of being greatly affected with trembles, milk-sickness," &c., and gives the following description of its topography:

---

*McCall, Western Jour. Med. and Phys. Sci. Vol. 3.
†Transylvania Journal, Vol. IX.

"The surface of this portion of the district is so flat and level as to constitute it a table land. The streams which flow from it towards the Scioto, the Little Miami and Mad river, have channels so little inclined, that when low, the water almost stagnates, while from the same cause when high, they overflow their banks. After lagging for a time in these nearly horizontal beds, they approach the deeply cut channels of the Scioto and Little Miami, when their currents acquire greater velocity, and before they reach their destination, several of them present rapids, some even cataracts. In proportion as this occurs, the diseases we are investigating diminish in frequency, so that in the actual vallies of the two principal rivers, they are seldom met with. In fact they may be said to be limited to the table land. Confining our attention at present to it, we may note several varieties of surface. 1st. Prairies, which are the beds of shallow lakes or ponds, which have been gradually filled up, and the greater part or the whole of the water evaporated. 2d. A kind of maple swamps, or wet wood lands adjoining to the prairies, and nearly or quite on the same level with them. 3d. Barrens, also contiguous to the prairies, but generally a little elevated above them, with a poorer and dryer soil, presenting the remains of an oak forest, which had been thinned out by fire from the prairies. 4th. Heavily timbered oak lands, nearly on the same level with the last. 5th. Scattered through these oak lands, small wet places and shallow ponds, called by the people "slashes."* Of that kind of rolling, dry and fertile surface, which is presented by the central and north-eastern counties of Kentucky there is none." Dr. Drake continues: "In our exploration of the district, whenever we traversed the

---

*This is probably a corruption of slush, itself, a corruption of sludge. Being in universal use in the district, we shall adopt it into this memoir.

prairies and adjoining flats, or barrens, or hill lands near the rivers, we could not meet with a single person, who had known the disease to occur, in those situations; on the other hand, whenever we came into or near the oak plateaus, every body, not only admitted its existence there, but equally admitted, that as far as he knew or had heard, the other varieties of surface did not produce it. Thus giving testimony against the salubrity of his own lands, and thereby affording satisfactory evidence of his sincerity. On the question, whether the cause is connected with the oak lands themselves, or the elm slashes which they embosom, we found some diversity of opinion, but a large majority fixed the mischief upon the latter, still further narrowing down the field of inquiry, if they should be correct."

From these extracts it must appear plain to all, that the reputed milk-sickness is not limited to any particular kind of country, but occurs in valleys, and on mountains, and hill-sides; on wet plains and dry ridges; and amid every geological formation, from the oldest lime-stone up to the coal series.

*Season.*—Let us next turn to see whether the testimony respecting the season of its prevalence is more concurrent. If the cause be a vegetable, the disease should prevail during the season of vegetation. If a mineral, it might appear at any season; and if it be a disorder induced by various causes, then it would appear whenever they were in operation. On this point Dr. Smydth observes: "The season at which milk-sickness most prevails is the autumn, after vegetation has been killed by the frost; and it has been observed to be more prevalent during dry seasons, when the streams are very low. In fact, I do not remember to have seen a case of it when the streams were flush. If it is ever seen in spring or summer, it is a rare occurrence. Sometimes we see nothing of it for two or three years, owing, no doubt, to the fact that the waters are kept flush by rains; but if an unusually dry season occur, the complaint is apt to prevail to a considerable extent."

The testimony of Dr. Armstrong is to the same effect. "We know," says he, "that the disease is more prevalent during a dry than a damp season of the year."

Dr. Seaton, who investigated this subject with much zeal in 1841, says, the people in the milk-sick regions "all united in declaring, that in very wet or rainy weather, they had but few cases of milk-sickness, either in persons or stock, but that during very dry weather, they suffer, winter as well as summer; and that when light rains succeeded to dry weather, it was always most abundant, until it was arrested by one good sweeping rain; which never failed to stop it forthwith." He adds: "In this section of Indiana, the milk-sickness is always feared in dry weather; but it is fearfully prevalent when small rains succeed a drought, and is arrested as soon as the earth's surface is well swept, or washed by a heavy rain."*

Dr. Sharp says: "It occurs at any season of the year, but is most common during the months of March, April, May, September, October, and November."†

According to the testimony of those who had observed the malady on the Miami river, Dr. Drake reports, that "it sometimes commences in July or before, but oftener in August, and continues till the approach of winter, when it generally but not always subsides."‡

When investigating this subject many years ago, the writer was assured by a number of those who were familiar with the disease as it prevailed on Goose creek, near Hartsville, Tenn., that it appeared about the latter part of May, and was most fatal early in June. The ex-

*Treatise on milk-sickness. By John S. Seaton, M. D.
†Western Jour. Med. and Phys. Sciences, Vol. 3.
‡Western Jour. Med. and Surg. Vol. 3.

perience of Dr. Thompson, however, was different; he says, "the season of the year at which the disease is most prevalent, is the fall, after vegetation has been mostly killed; about the time of white frosts, and from that time till the cold of winter becomes constant. The next worst time is about the appearance of vegetation in the spring; though cattle sometimes suffer if the winter is mild, after unusually warm spells, in December, January, and February."*

Of six cases related by Dr. Sharp, two occurred in July, two in May, and one on the 26th of April. The date of the sixth, which was chronic, is not given.

Dr. White says: "The disease is most severe during the dryest season of the year, be that early or late. In a wet season, such as the last, we see but little of it. But one case occurred within our knowledge in the course of last summer, which was brought on by eating dried beef, that had been slaughtered the preceding fall."†

Dr. M. L. Dixon published an essay on the subject of Milk-sickness in the Transylvania Journal of Medicine,‡ in which he remarks, that in his practice about Winchester, Tenn., he had seen much of the disease. After stating that the places in which it appears are "low, fertile, marshy districts, as in the deep, rich valleys among mountains, or near the margins of rivers and creeks," he goes on to say, that the affection "is most prevalent from May until November, in all which season," he continues, "I have seen numerous cases. During very mild seasons," he adds, "when vegetables continue verdant throughout the autumnal months, the case may be different; but I have no reason to believe that any case ever arises after winter has fairly set in. If the complaint is developed in mid-winter, it is most probable," he thinks, "that the

*Transylvania Jour. Med. Vol. I.

†Vol. IX.

‡Vol. VI.

seeds of it were sown during the season of vegetation;" for according to Dr. Dixon as well as some other writers on Milk-sickness, "it is one of the peculiarities" of this anomalous disorder, "that the poison occasionally lies long dormant in the system, and only becomes active when excited by some indiscretion, or violent bodily exertion."

It would be unprofitable to adduce more testimony on this head. Nothing could be more variant than the experience of the different observers; nor can it fail to strike any one who reads attentively the various accounts of Milk-sickness, that each writer is insensibly biased by his prepossessions as to the cause of the complaint. Those who attribute it to a vegetable poison are anxious to find that it does not prevail after the frosts have put a stop to vegetation; whilst the advocates of a mineral poison insist, that it is independent of the seasons; and all are compelled to admit that, because the weather is mild, or for some other reason, cases do occasionally occur in mid-winter.

*Cause.*—We will now turn to the cause, and, as on the other points, quote from the various writers who have had experience in Milk-sickness. As far as I have been able to inquire, all who have recorded their observations on this subject ascribe the disease to a vegetable or a mineral poison, and the writers are about equally divided between the two theories.

Dr. Smydth is clear, that the cause of the disease in cattle, which originates Milk-sickness in the human subject, is one of the mineral poisons. He thinks that it may be arsenic, cobalt, zinc, or lead, and that streams of water running through it may thereby become impregnated to such an extent, as to produce its specific action upon the animals that drink it. Dr. Armstrong, Dr. Seaton, Dr. White, Dr. Shelton, and some others, incline to the same view. Dr. Smydth relates the following instances to show that the complaint cannot originate in a vegetable poison: "A gentleman living near Bloomfield had a blue-grass pasture containing about five acres, upon which no vegetation but the grass was to be seen growing. The pasture was enclosed in a good fence; a small stream of water ran through it. A horse and two or three milch-cows grazed upon the pasture. During a very dry autumn, a few years since, the owner was attacked with Milk-sickness, and while he was sufferiug with the disease one of the cows was seized with the "trembles" and died. A dog having eaten of the flesh of the cow was also attacked with "trembles" and died. The horse was affected about the same time in a similar manner, but recovered.

"Mr. B., living in the same neighborhood, had occasionally experienced Milk-sickness in his family, and his wife had fallen a victim to the complaint. Several of his cows and other domestic animals died of trembles. Suspecting that a spring from which he had been in the habit of using water might be the cause of his troubles, he removed his dwelling a short distance to the neighborhood of another spring, and had the former one enclosed in a fence so as to keep his live stock away from it. Several years have now elapsed since the change was made, and no more cases have occurred in his family or among his cattle."

Dr. Armstrong mentions the following instance as confirmatory of the same position: "In the county of Martin, Indiana, a farmer who had lost several domestic animals and one member of his family with the disease, purchased a cow in an adjoining county where Milk-sickness was not known. She was turned into a naked lot and fed upon Indian corn meal. There was a spring of water in the lot from which the cow obtained drink; but there was no green vegetable growing in the lot. In the confidence that she could not get any poison, the family used her milk freely. Not long after she was confined in this lot, it was observed that she walked sluggishly, and began to tremble when forced to move. About this time, one of the family, a daughter, was seized with Milk-sickness and died. The cow also died shortly after."

Dr. Armstrong proceeds to relate, that a physician, who attended the case just recited, found a suspicious looking stone in the bottom of the spring in the lot, and that, taking it with him home and exposing it to the air, he found, after a few weeks, that it had "crumbled into a grayish powder." Many cases, he continues, might be cited, in which cows feeding upon blue-grass pastures destitute of all other kinds of vegetation, have become afflicted with the trembles and died. In all such cases, he thinks, minerals similar to the one just spoken of, are to be found in the neighborhood of the streams affording water to the cattle.

Dr. Seaton remarks: "In Lawrence county, on the waters of Beaver creek, one of the tributaries of East White river, I made the most of my observations. I found the whole earth there to be full of the different specimens of iron ore, combined with the hard, bastard limestone, yellow and red sandstone, indurated clay or "soapstone," some shale or slate, blue clay, stonecoal, &c. We were informed by several gentlemen residing there, that lead ore had been found by different persons, also, on the surface of the earth. But the iron ore is abundant, the Milk-sickness equally plenty, and numerous plantations are entirely depopulated, many having died of the Milk-sickness, while others, after having lost more or less out of their families by this ailment, moved away, leaving their landed possessions in that section, free to any who may wish to occupy them,

"The unanimous belief in that section is, that the disease is caused by some mineral substance; and I think it impossible for any man to pass through that region, and arrive at any other opinion. The people speak of the poison springs there, as familiarly as we do of any other known subject. We visited many springs that had been fenced in or abandoned, and nearly all of them, we found the white pyrites in abundance."

Dr. Sharp says,* that in the neghborhood of Hartsville, Tenn., where Milk-sickness has been endemic since the early settlement of the country, "there has never been an instance of the complaint in any species of live stock while they are confined to an old well-cultivated pasture; nor have any persons ever been affected by using the milk or flesh of cattle thus pastured. "But," he adds, "I have seen the disease make its appearance in families shortly after adding a piece of woodland to their pastures, and disappear on separating the timbered land from the old fields."

From all that I could gather, in 1828, from those who had resided in the milk-sick region, on Goose creek, I felt authorised to draw the following picture:

"The cattle of one family are permitted to frequent the woods; they return home at night, apparently in health, it may be; the members of the family partake of the milk, and are soon attacked with sickness of stomach, vomiting, and other symptoms of gastric derangement, and perhaps some individual of it perishes. The calf, meanwhile, is taken with trembling, violent thirst, and rigidity of the extremities, of which it dies. The cow, all the time, which imparted the disease may be, to all appearance, in good health—an appearance which severe exercise, or simply retaining her milk, shews to be delusive. In returning home some of the cattle have perhaps died at the first stream on their way after drinking copiously; whilst others, scarcely able to reach home, manifest unequivocal symptoms of disease, teaching the family to beware of their milk.

*Western Jour. of the Med. and Phys. Sciences, Vol. III.

'While this is going on in one family, another, living in the same neighborhood, inhaling the same atmosphere, drinking the same water, and faring upon the same diet, but with the precaution of confining their cattle to cultivated pastures, remains healthy. This was a circumstance of every day occurrence in the early times of Goose creek, when the inhabitants were more in the habit, than at the present day, of permitting their milch-cows to wander abroad. It is a fact of perfect notoriety, that persons living in the neighborhood where the poison prevails may indulge in the use of milk, butter, and fresh meat, and yet remain free from disease, provided the cattle be enclosed in clover, or other grass pastures. But if they be suffered to run at will, or even if the pasture-ground be enlarged by taking in more woodland, disease, and death, often, are the consequence. Upon again excluding the woodland, which contained the poison, and confining the cows to the soil set with grass, health may be, as in many such instances it has been, again restored. Dr. Thompson's letter to me furnishes a case directly in point. 'Robert M'Neely, his wife, and four children died within a week, under circumstances which left no doubt on the minds of any, that their death was the result of using poisoned milk. He had lived a number of years at the same place, and enjoyed good health, except that he was subject to attacks of humoral asthma. Having escaped so long he became careless, and during a busy season of hay-gathering his cattle got out, and were not attended to for several days. The first milk taken after their return from the woods produced all the mischief. The effect followed its use almost immediately. All the family died except the two youngest children—one an infant at the breast, and they were saved with difficulty.'"*

---

*Transylvania Jour. Med. Vol. I.

Dr. McCall states positively, that, on the Yadkin river, the disease "could always be eradicated by cultivating the soil."

Dr. Dixon says: "The cultivation of the soil has been known to eradicate the cause, which, if anything but a vegetable, it is not easy to understand." He adds: 'In many of the tracts of country in which the disease prevails, there are no evidences of any mineral substance whatever."

Dr. Winans, of Jamestown, Ohio, assured Dr. Drake, that the disease never prevails in that region "except in and near the heavily timbered oak lands; that it is not generated in the prairies, flats, barrens, or hills, and that *it ceases when the forest is cut down.*"

A farmer made the following statement to Dr. Drake: "About twenty years ago, being a grazier, he purchased about one hundred head of cattle from his neighbors, and separated the younger and more infirm, to the number of about thirty, from the rest, and turned them into a new meadow, formed out of elm and maple slashes. Most of the trees had been felled, and the ground harrowed, but not plowed. The sown grass had taken root two years before. The remaining seventy, he placed on the opposite side of a narrow lane, in a wood-pasture, the surface of which was precisely of the kind which the meadow *had* been. The whole were salted at the same time, and watered from the same well. Nothing was fed to any of them. On a certain day, in salting them, they all seemed perfectly well in both fields; but the very next day, between thirty and forty, or about one half of those running in the woodland pasture, were found to have the trembles. He immediately turned all that remained well, into the meadow, where four or five of them afterwards sickened with the same disease. Those which were ill, he turned into a corn field, and fed them with unripe indian corn,

Nineteen of them died, while not an individual, originally placed in the meadow had the disease."

Dr. Drake infers from all the facts he was able to collect on the subject, "that clearing and cultivation, even girdling the trees, harrowing the ground and sowing it with grass seed, destroys or renders inactive the cause (of trembles) whatever it be;" that the disease in Ohio is much less prevalent than formerly; and that there has been a regular decrease of it, as the forest was transformed into arable land, and stock were kept within enclosed meadows."

Dr. Shelton affirms, that Milk-sickness "is generally met with in the immediate vicinity of mines," but admits that there are exceptions to this rule. "In most parts of Tennessee," according to his observation, "it prevails in the neighborhood of iron ore, which is mixed with a variety of other metals." He states that it is common near the Minor Breton lead mines, in Missouri; and adds, that "a point exists in Tennessee, at which several persons and many domestic animals have lost their lives from this poison. It is a tract of country surrounding some old lead mines, which were purchased at a high price by a company from Virginia, but abandoned on account of the difficulty of working the ore, owing, as it is supposed, to the presence of other metals." Dr. Shelton, however, admits that the disease ceases to appear if the pastures of the cattle be sown with clover.*

In proof of the mineral origin of the disease, Dr. White relates the following circumstances:

"A respectable farmer informed me, that his cattle had been in the habit of frequenting a pasture ground in company with his neighbor's, on the opposite side of a creek from him. In returning home, his cattle were obliged to

*Trans. Jour. Med, Vol. IX.

cross the creek. For many years not a case of the disease appeared among them, while his neighbor lost some forty or fifty head during that time. The animals of the latter did not cross the creek, but drank at another stream. Both herds ranged the same woods, and fed upon the same herbage. It is presumable, therefore, that the disorder was produced by the water ; and in confirmation of this opinion, this individual farther stated to me, that suspecting a spring at which his cattle drank to be the origin of the evil, he set to work felling trees around it, so as to exclude his stock from it, and that afterwards they suffered no more with the disease for several years. At length, however, it recurred again, and on examinntion it was found, that the spring had become accessible from the decay of the timber. The enclosure being repaired, and the cattle shut out from the water, the disease a second time disappeared.

"Another farmer of respectability told me that a number of his sheep, kept upon a grass lot on account of wolves, had died with the symptoms of milk-sickness. During every season several had died for a series of years, until his flock was nearly destroyed. He at last began to suspect a dripping spring in the lot, as the source of the mischief, and accordingly took measures to secure the animals against it, whereupon the disease ceased. In this lot it is not probable that there existed any vegetable to which the affection could be attributed."*

What is to be made of these contradictory statements? Who will attempt to reconcile them? According to one report, cows confined to a bare lot and fed with corn meal, have contracted the disease and communicated it to whole families through their milk. According to others, the milk of cows in the very midst of the regions where the

---

*Transylvania Jour. Med. Vol. IX.

poison is supposed to abound may be used with safety, provided the animals be kept upon cultivated pastures. We have it upon the most unquestionable authority, that the disease has been contracted in localities where the stony strata are concealed by a deep alluvium and no minerals appear on the surface; and we are assured by others that it has originated where there were no vegetables to produce it. If we are to receive both observations as correct, then we must conclude that the complaint is independent alike of minerals and vegetables. If it is confined to no season, I can not help believing that the vegetable kingdom must be exonerated; and if it has been long endemic in districts barren of minerals, how are we to get over excusing the mineral kingdom?

At the same time, there are difficulties attending this view of the subject, as insurmountable ones certainly surround either of the hypotheses under discussion. The question still recurs—*what is Milk-sickness?* Upon what morbific agent do the symptoms, known as that affection in the human subject, and as "trembles" in the lower animals, depend? The phenomena must have a cause. The disease is an entity; greatly dreaded, indeed, and very formidable, in many places. To be convinced of the truth of this, one has only to read the foregoing extracts. Its victims among our species have been numerous, and vast numbers of our domestic animals have perished by it. What is it? It bears some resemblance to mineral poisoning, but it is a very imperfect resemblance; a thousand objections might be urged against its being an effect of arsenic, lead, or any known mineral. This is the most untenable of all the theories started on the subject. It will not bear the slightest examination. It wants every element of a plausible theory. And whether any known plant is capable of producing all the effects described as resulting from the unknown poison, the reader will be better able to judge after he has weighed what follows.

*Symptoms and Character.*—The first account of Milk-sickness anywhere published was given by Dr. Drake, in 1810, in his "Notices concerning Cincinnati." Dr. Barbee, of Virginia, returning from the Mad river country, in the spring of 1809, communicated to Dr. Drake some facts in respect to it, and from his description and the narrations of others who had experienced the disease in their own persons or in their families, he wrote the following notice of it:

"It almost invariably commences with general weakness and lassitude, which increase in the most gradual manner. About the same time, or soon after, a dull pain, or rather soreness, begins to affect the calves of the legs, occasionally extending up to the thighs. The appetite becomes rather impaired, and in some cases nearly suspended; sensations of a disagreeable kind affecting the stomach; upon taking a little food, however, a greater disposition for it is generated, and more agreeable feelings are introduced throughout the whole system. Intestinal constipation, in this, as in the subsequent periods of the disease, exists in a very high degree. A strong propensity to sleep occurs, and according to Dr. Barbee, the pulse is "full, frequent, round, and *somewhat* tense, but regular." During this stage, exercise of any kind is highly detrimental, and if persisted in, soon inducing loathing and nausea at the stomach. If the patient repose, upon first experiencing these symptoms, they generally cease, and he is allowed a longer exemption from the *vomiting* that awaits him. Sooner or later, however, that symptom almost invariably succeeds the predisposition we have described, and either proves fatal in one, two, three or more days, or leaves the patient in a most exhausted state, from which he recovers only to sustain, at no distant period, a repetition of the same attack.

"The matter ejected is sometimes bilious, but much oftener sour, and so acrid, that its action on the throat, in one case, (which proved fatal) was likened to that of boiling water. Towards the close of mortal cases, it is occasionally very dark colored, so that it has been compared to that very convenient and fashionable object of similitude—coffee grounds. At this time the intestinal constipation is very great. Mr. Snodgrass knew one patient in whom it continued for nine days, throughout which he took no food whatever, and vomited during six of them. After such an attack, the propensity of it is destroyed, and an uncommon degree of watchfulness is produced. The patient remains languid, and his face and person generally become rather tumid. His skin is cool, palish, and frequently affected with clamminess. He has a disagreeable burning sensation in his stomach, and hot eructations are very troublesome. The thirst is considerable. The breath is peculiarly disgusting, even loathsome. The appetite is generally poor; and the inclination to costivness remains. These symptoms often continue for several months, during which the patient experiences frequent returns of the vomiting. But at length, more especially upon the approach of winter, they gradually wear away, leaving the patient considerably worse than they found him, and liable to a fresh attack the ensuing summer.

"Nothing like *regular* periodical exacerbations is observable in this disease; no chilliness occurs; the color of the skin and eyes does not deviate widely from that of health, and gives no striking indication of bile; there is no pain in the region of the liver, nor in the shoulder; it does not terminate in dropsy; nor are there any symptoms which bespeak it a disguised or anomalous intermittent. It however prevails (though not exclusively) in aguish situations, and intermitting diseases are thought to have declined since its appearance.

"It affects all ages, conditions, and both sexes, indiscriminately; except probably very young children. They however, are not wholly exempt from it. Emigrants are not peculiarly liable to it. It was first observed in the summer of 1806, and is thonght annually to extend its geographical range, and to become more intense."

Dr. Sharp thus describes the symptoms presented by some cases which he treated in the neighborhood of Harts-ville:

"A burning sensation in the stomach was first felt. It was moderate for a day or two, and not attended with any vomiting or pain. On the third day after their respective attacks, each complained of severe burning at the stomach, and vomited frequently. Obstinate constipation of the bowels attended both cases. The pulse was small, thready, and a little accelerated; and the heat of the extremities considerably below the common temperature. The tongue was tremulous and covered with white mucus. Restlessness and great anxiety prevailed in the early stage of the attack.

"A constant and ungovernable thirst was present; and when water was drunk it only served to allay the thirst for a few moments; being quickly ejected and the pain and vomiting increased. There was no pain in the region of the liver, the spine or the head.

"The stomach and bowels seemed to be the seat of the disease, and great prostration of strength soon followed. These pains with occasional hiccuping continued with unabated violence, for about twenty-four hours, from the time the vomiting commenced. The vomiting and pain then began to subside, and the hiccups became frequent. Inspiration was performed slowly and with much difficulty, and as the patients became less restless, and the anxiety abated, stupor began to prevail in the same ratio, till they became completely insensible."

All the writers who have attempted to enumerate the symptoms of Milk-sickness agree in the vomiting, the intense burning sensation in the epigastrium, and the obstinate constipation, as being attendants upon cases of any duration; but instances of sudden death snpposed to have been caused by this disease are on record where all these phenomena were absent. In fact, while the descriptions of the writers generally agree when they attempt a portrait of the disease, one can not help remarking, as he reads the various papers on the subject, that many cases are set down to its account which want all its features. Thus, the dog, which vomits as easily as man, instead of exhibiting the symptoms that appear in the human subject, is represented as faltering in the chase, or dropping down and suddenly expiring. And whilst, as a general rule, the disorder is described as coming on slowly and insidiously, cases are mentioned of children suddenly attacked while sucking their mothers, and men dying at the table from the effects of the poison! I will proceed to give some of these instances more in detail, which will afford an idea of the incongruous qualities attributed to this affection.

Dr. Lewis relates an instance in which seventeen members of a family were attacked, almost simultaneously, with milk-sickness; but fails to describe the symptoms. Two individuals died. The cattle had been in a milk-sick region, and many of them were affected with trembles at the time the family were sick. Dr. Lewis also gives the following, on the authority of a fellow-student:

"The stock of a Mr. Townsend, who lived near Winchester, Tennessee, strayed some distance from home in the fall of 1819. He was compelled to drive them through a milk-sick district of country, in returning. One of his steers complained slightly before getting home, but after resting, it became to all appearance well. Soon after his arrival at home, Mr. T. butchered this steer, and sent it to market, reserving only a small portion for his family. On the same day that the beef was butchered, a portion of it was made into steak, of which himself and family ate heartily. Mr. T. expired at the table while eating, his wife soon after; and in a few days one of his daughters. Every individual, who ate of this beef, was more or less violently attacked, with milk-sickness."*

Dr. Dixon says, "cattle that have received the poison may appear well for weeks, and exhibit it upon being made to travel or labor. And while in a condition thus apparently healthy, the milk or flesh of an animal may be eaten, and the person thus eating may contract the disease. The same peculiarity marks it in the human subject. Where all the family have subsisted upon the same articles of diet, one will suffer and another remain well, because they have not been subjected to the same exciting causes. Violent exertion, or whatever else is calculated to weaken the system, has been known to excite it in persons predisposed to it."†

A farmer informed Dr. McCall, that "one of his dogs, having partaken of diseased beef, ran after a rabbit across the field, and then fell down and died; and that another dog died in the same way, whilst in pursuit of a hog."‡

A Mr. Miller, living in the reputed region of milk-sickness on Goose creek, Tenn., gave Dr. McCall the following certificate:

"I have been in the habit of keeping up my stock for twenty years past. About four years ago my sheep were turned out of the pasture, and soon after seven or eight died. Not supposing they were poisoned, I did not burn their carcases, and my hogs eat of them. These hogs died and were destroyed, except one which I did not find in the lot. This body was found to be picked by the chickens, which chickens, as I believe, caused Milk-sick-

---

*Inaugural Dissertation, by John T. Lewis, M. D., Transylvania Jour. Med. Vol. 2.

†Transylvania Jour. Med. Vol. 6.

‡Western Jour. Med. and Phys. Sci. Vol 3.

ness in myself, my wife, and three children, by using them as diet. My reason for believing so is, that we had the true milk-sickness, and had eat no other fresh meat; and my son who eat none of the chickens, or soup, was not sick. My cattle had not been out of the old lot, and he as well as the rest of us used their milk. What, however, acted as convincing proof to my mind was, that so long as we used chicken soup as diet, in our sickness, we all grew worse: till suspecting the fowls for being the cause, we abstained from using them. My wife died; and I still feel the complaint after taking exercise.*

JOHN MILLLR."

Upon this Dr. McCall remarks—"Whether or not Mr. Miller be right in his conjecture of the cause of sickness in his family, one thing it seems is ascertained, that the poison can in three animals successively generate the disease, by only using the flesh of each one as it dies."

Dr. Sharp says—"Young calves die with the usual symptoms, whilst their mothers appear in good health. And the flesh of such calves will communicate a similar disease to other animals feeding on it."† Dr. Sharp further states, that "he has never failed of curing his patients, after their bowels had been freely and early evacuated."

In the paper to which reference has been made, I stated upon the testimony of persons who professed to have witnessed examples of the kind, on Goose creek, that calves which had perished from sucking an affected cow, imparted the disease to dogs and buzzards feeding upon their flesh. In that paper, Dr. Thompson has a statement to the following effect:

"A farmer's cows, four in number, broke out of their

---

*Western Jour. Med. and Phys. Sci. Vol. 3.
†Ibid.

enclosure, and were in the Mill-stone knobs about thirty-six hours. When driven home some of the milk was drawn from them and set aside that its appearance on standing might be observed; the calves were permitted to suck the rest; they were hungry, the udders of the cows full, and consequently, their repletion was copious. One of the calves was taken sick while sucking and died in a few hours. All the others died in a day or two. Three of the cows died; one which was sucked by two calves, both of which died, recovered, after severe disease, in the course of which all her hair dropped off. The milk which was set by was given to several domestic animals, as cats and dogs—all of which died, if I am not mistaken; some I am confident did, and all were diseased."*

Dr. Shelton affirms that the disease may be prevented in cattle by giving the animals an abundance of clover. This experiment, he says, has been repeatedly tried in Tennessee and Indiana. "Fields known to have contained the cause of the disorder, have been freed from it in repeated instances, by setting it well in clover; and it has again appeared on the clover being ploughed up, and the field sowed in grain."†

At the risk of being somewhat tedious, I have mnltiplied quotations on this part of my subject, in order that the reader may have at one view the various accounts of the different writers respecting the operation of the reputed poison of milk-sickness. And, certainly, he will exclaim, if there be such a poison, it is a strange one: a poison so variable in its action that some members of a family are destroyed by it, while others take it with impunity; that may lurk in the system for weeks without

*Transylvania Jour. Med. Vol. I.

†Ibid Vol. IX.

manifesting itself, and again kill a man while eating food containing it at table; that may be prevented by clover, and yet be transmitted in all its force through the flesh of the ox to the dog, and from the dog to the carrion-crow; so virulent as to induce fatal disease in the calf, and yet not visibly affect the cow affording the milk, or cause sudden death in a man, while the ox that supplied the poison was still able to travel and only slightly indisposed; a poison that may render the flesh of sheep so noxious as not only to kill the hogs devouring their carcases, but so affect the chickens picking about the dead bodies of the hogs as to make soup prepared from their flesh poisonous, the chickens, all the while, running about apparently in good health! If this is not marvelous, then I confess the history of poisoning contains no marvels. What are we to conclude? Of course, we are to reject the case in which the dried beef was charged with being the cause. We must reject Dr. Lewis's case of the man dying at table from the effects of the poison. We must also reject Dr. McCall's case in which the disease was ascribed to the diet of "chicken soup." We can not help receiving as doubtful, the alleged prophylactic virtues of red clover. And, upon a review of the whole matter, the conclusion to which all the testimony on the subject has brought me is, that we, who have written upon milk-sickness, have been egregiously imposed upon by careless and incompetent observers. We have been very credulous. We have believed many things which, when we come to investigate them, we find to be impossible. In illustration of the credulity exercised on this subject, I will quote a case referred to by Dr. McCall as proof of the transmissibility of a poison through the milk of the mother. The case, one of *snake-bite*, is recorded in the third volume of *Coxe's Medical Museum*, and is as follows:

"Some time in the summer of 1901, the wife of Mr.

Alfred Bremin, in the town of Baintrim, Luzern county, Pennsylvania, was bitten by a rattlesnake. She was then in the fourth month of pregnancy. After some considerable degree of the common consequences of such an accident had occurred, she at length recovered. At the full time of delivery she was safely put to bed. The child was apparently healthy; but immediately after allowing it to suck, it assumed the hues of a rattlesnake, swelled very much and soon died. She then procured a puppy which died in two days of the same symptoms, and in succession one puppy and three lambs shared the same fate. Another puppy was procured which discovered but little of the symptoms and did not die. In 1803 she had another child, and no disagreeable symptoms resulted from the use of her milk."

Now, it can hardly be necessary to remark, if the venom of the snake had been taken directly into the stomach of the child instead of being swallowed with its mother's milk, no such effects as those described would have followed. However virulent this poison may be in the bloodvessels of an animal, it has been proved to be innoxious received into the stomach. The case is in keeping with many of the reputed cases of milk-sickness.

That the affection known in our domestic animals as "trembles" may depend upon some specific poison which certain localities afford, is possible; but it is not possible that any poison can produce all the consequences charged upon the poison of milk-sickness. It is possible that there may be a plant which affects cattle in this way; but no plant can be harmless to some members of a family and poisonous to others. It is impossible that the train of symptoms given as constituting trembles, can originate in metallic poisoning. They are not the symptoms of such poisoning. Who can believe that the flesh of cows may be so saturated with arsenic, or copper, or the salts of mercury, as to kill hogs, dogs, and buzzards? Who does not know that it is nearly impossible to kill hogs or dogs by arsenic?

But granting that there is a plant to which the disease in cattle is referable, we must admit that it is not among those the properties of which have been ascertained. It is no common plant. It must be one of the most extraordinary of all vegetable productions.

Nor does it seem to my mind clearly made out, that there is any connection between the milk-sickness of the human subject, and the trembles of cattle. True, the former affection generally follows the use of milk, butter, or beef; but what disease does not? These articles of diet are in almost universal use, and it can not therefore be deemed remarkable that in every case of milk-sickness the subject can be shown to have partaken, sometime previously, of some of these things. In some instances the attack has been ascribed to unsound beef, when it has turned out that only a few of all those who had eaten of the beef were affected. In many more it has been referred to the milk of animals which, at the time it was used, had no appearance of being diseased. This may show how loose and untrustworthy have been the observations relative to this complaint.

Upon the whole, is it not probable that the coincidence of trembles and milk-sickness has been merely accidental? Is it not probable that other kinds of poisoning have been mistaken for the effects of this specific poison? and is it not certain that cases have been set down as milk-sickness, which had no connection with milk, and as little, probably, with the supposed cause of that affection? The case of Mr. Miller, reported by Dr. McCall, and the case referred to by Dr. White, as induced by dry beef, are clearly of this character.

The various accounts of this disorder nullify each other; and the mind is left in extreme doubt whether there is anything *specific* in milk-sickness. What the disease is which has received this name, is a matter worthy of further investigation.

Louisville, Ky., April 17th, 1852.

